# Molecular and Epidemiological Characteristics of Carbapenemase-Producing Klebsiella pneumoniae Clinical Isolates in Japan

**DOI:** 10.1128/mSphere.00490-20

**Published:** 2020-10-21

**Authors:** Shinsuke Yonekawa, Tomoki Mizuno, Ryuichi Nakano, Akiyo Nakano, Yuki Suzuki, Tomoko Asada, Ayako Ishii, Naoki Kakuta, Kosuke Tsubaki, Sayaka Mizuno, Miho Ogawa, Hisakazu Yano, Kei Kasahara, Keiichi Mikasa

**Affiliations:** a Center for Infectious Diseases, Nara Medical University, Kashihara, Nara, Japan; b Department of Microbiology and Infectious Diseases, Nara Medical University, Kashihara, Nara, Japan; c Department of Bacteriology, BML Inc., Kawagoe, Saitama, Japan; Antimicrobial Development Specialists, LLC

**Keywords:** *Klebsiella pneumoniae*, carbapenemase, hypervirulent clones

## Abstract

Carbapenemase-producing *Enterobacteriaceae* represent a serious public health threat worldwide. Carbapenemase genes, harbored on a transferable plasmid, have been isolated globally with distinct geographical features. Klebsiella pneumoniae, included in *Enterobacteriaceae,* also produces carbapenemase and often shows hypervirulence. Overlapping carbapenem resistance and hypervirulence in K. pneumoniae have been reported, but such strains have not yet been found in Japan. Here, we screened 104 carbapenemase-producing K. pneumoniae isolates collected from 37 hospitals and outpatient clinics in Japan between September 2014 and July 2015. PCR and DNA sequencing demonstrated IMP-1 in 21 isolates and IMP-6 in 83 isolates, 77 of which coharbored CTX-M-2. Most of the isolates showed low MICs toward imipenem and meropenem but high MICs toward penicillin and cephalosporins. Conjugation experiments with an Escherichia coli J53 recipient showed that most of the plasmids in IMP-6 producers were transferable, whereas only one-half of the plasmids in IMP-1 producers were transferable. PCR-based replicon typing and multiplex PCR identified five isolates belonging to the CG258 non-*tonB79* cluster and no isolate belonging to the CG258-*tonB79* cluster or sequence type 307 (ST307). Four K1-ST23 isolates, 10 K2-ST65 isolates, and 7 K2-ST86 isolates were detected that harbored virulence genes. The resistance genes in 85 isolates were transferable, but the virulence genes were not transferred. These results demonstrate the acquisition of IMP-type carbapenemase genes and CTX-M-type genes among hypervirulence isolates in Japan, warranting further attention and countermeasures. In this study, we have determined the molecular characteristics and epidemiology of IMP-6 producers that coharbored various CTX-M genes in Japan.

**IMPORTANCE** Carbapenems serve as a last resort for the clinical treatment of multidrug-resistant infections. Therefore, the rapid spread of carbapenemase-producing strains represents a serious public health threat, further limiting antibiotic choices. The current findings of hypervirulent carbapenemase-producing Klebsiella pneumoniae clinical isolates in Japan demonstrate the potential broad spread and transfer of these genes, necessitating close surveillance.

## Abstract (precis)

Carbapenems serve as a last resort for the clinical treatment of multidrug-resistant infections. Therefore, the rapid spread of carbapenemase-producing strains represents a serious public health threat, further limiting antibiotic choices. The current findings of hypervirulent carbapenemase-producing Klebsiella pneumoniae clinical isolates in Japan demonstrate the potential broad spread and transfer of these genes, necessitating close surveillance.

## INTRODUCTION

Carbapenems such as imipenem and meropenem are the most potent agents available for treating infections with Gram-negative bacteria owing to their stability to the majority of β-lactamases and the high rate of penetration of bacterial outer membranes (1). Therefore, carbapenems are among the most useful antibiotics for treating patients with severe infections. In Japan, carbapenem-resistant *Enterobacteriaceae* (CRE) remains rare. According to the 2019 National Surveillance in Japan, the prevalence of CRE (carbapenem resistance is defined as either resistance to meropenem [MIC ≥ 2 μg/ml] or resistance to both imipenem [MIC ≥ 2 μg/ml] and cefmetazole [MIC ≥ 64 μg/ml], based on susceptibility tests) was 0.33% (2). Another study in Japan found that the isolation frequency of carbapenemase-producing *Enterobacteriaceae* was low (0.35% [17/4,875] of clinical isolates of *Enterobacteriaceae*) (3).

However, many clinical isolates have been reported to be resistant to carbapenems due to the production of carbapenemase worldwide. In particular, there have been increasing reports of *Enterobacteriaceae* carrying transferable carbapenem-resistance genes such as those encoding the carbapenemases IMP, NDM, VIM, Klebsiella pneumoniae carbapenemase (KPC), and OXA-48 (4). Most of these carbapenemases confer resistance to not only carbapenems but also other β-lactams. These enzyme-producing bacteria are developing multidrug resistance, thereby displaying resistance to other antimicrobial agents such as aminoglycosides and fluoroquinolones, which limits the antibiotic of choice available for antimicrobial therapy in some cases, becoming a serious issue in clinical practice. We previously reported that the IMP-type was the predominant carbapenemase among Escherichia coli isolated in Japan, and IMP-6 was predominant among the IMP-type carbapenemases (5). However, the predominant carbapenemase among Klebsiella pneumoniae isolated in Japan has not yet been clarified.

K. pneumoniae is a frequent cause of nosocomial infections and has also been recognized as the cause of life-threatening and severe community-acquired infections, including pyogenic liver abscess, pneumonia, and meningitis (6). Multilocus sequence typing (MLST) of K. pneumoniae involves sequencing of defined regions in seven housekeeping genes (*gapA*, *infB*, *mdh*, *pgi*, *phoE*, *rpoB*, and *tonB*) (7), and K. pneumoniae isolates belonging to clonal group 258 (CG258) are the most common carbapenem-resistant isolates reported worldwide (8). Moreover, the isolates belonging to sequence type (ST)23, ST65, ST86, and ST375 show characteristics of hypervirulence (6, 8), and hypervirulent capsular K1 and K2 isolates, especially K1-ST23, K2-ST65, and K2-ST86, have been reported in Japan (9). Analysis of the K. pneumoniae MLST database (https://bigsdb.pasteur.fr/klebsiella/klebsiella.html) revealed a unique *tonB* allele (*tonB79*), which has been found only in strains typed as CG258 (CG258-*tonB79* cluster) to date. The global spread of carbapenemase (especially KPC)-producing K. pneumoniae has also been linked to CG258 as the single genetic lineage. Isolates belonging to the CG258-*tonB79* cluster, including ST258 and ST512, have been isolated in Europe, the United States, and China (6), whereas strains of the CG258 non-*tonB79* cluster such as ST11 have mainly been isolated in China and were found to harbor various carbapenemases, including KPC and NDM (10). Moreover, emergence of the carbapenemase-producing K. pneumoniae (CPKp) clone ST307 was recently reported in Europe, Africa, and Korea (11–13). ST23, ST65, and ST86 isolates were found to harbor virulence genes such as *rmpA*, *rmpA2*, *iroN*, and *iutA* at a high frequency (6). Thus, carbapenem-resistant STs and hypervirulent STs appear to belong to different groups in most cases. Although carbapenemase and virulence genes are located in a large plasmid and are transferable and K. pneumoniae isolates with dual properties of carbapenem resistance and hypervirulence were reported in other countries (14), they have not yet been reported in Japan.

In this study, we analyzed the national epidemiological and molecular characteristics of clinical isolates of CPKp and showed the dissemination of hypervirulent isolates among CPKp in Japan.

## RESULTS

### Characteristics of resistance genes in CPKp.

Among the 104 CPKp isolates obtained from 37 hospitals and outpatient clinics in Japan from September 2014 to July 2015, 83 isolates were found to harbor *bla*_IMP-6_ and 21 harbored *bla*_IMP-1_ (Table 1). No isolate harbored any other carbapenemase genes.

**TABLE 1:** Resistance genes, incompatibility, and transferability of carbapenemase-producing Klebsiella pneumoniae isolated in Japan

Resistance gene	No. of isolates	Plasmid incompatibility (no. of isolates)	No. of transferable isolates	Avg transfer frequency
IMP-1	21	FIIAs (1); HI2 (3); N (2); N, A/C (10); NT (5)	9	8.3 × 10^−5^
IMP-6	6	N (3); NT (3)	4	9.0 × 10^−4^
IMP-6, CTX-M-2	72	N (71); N, HI2, FIIAs (1)	67	3.2 × 10^−4^
IMP-6, CTX-M-2, CTX-M-15	1	N (1)	1	4.1 × 10^−5^
IMP-6, CTX-M-35	3	N (3)	3	5.3 × 10^−4^
IMP-6, CTX-M-65	1	N (1)	1	1.6 × 10^−7^
Total	104	FIIAs (1); HI2 (3); N (81); N, A/C (10); N, HI2, FIIAs (1); NT (8)	85	3.8 × 10^−4^

Among the 83 IMP-6 producers, 77 isolates coharbored the CTX-M gene (72 with CTX-M-2, one with CTX-M-2 and CTX-M-15, three with CTX-M-35, and one with CTX-M-65), and six isolates did not harbor a CTX-M gene. In contrast, none of 21 IMP-1 producers coharbored any CTX-M gene.

### Antimicrobial susceptibility profiles of CPKp.

The results of antimicrobial susceptibility testing with the agar dilution method are shown in Table 2. Most of the isolates showed low MICs toward imipenem and meropenem, whereas many isolates showed high MICs toward penicillin and cephalosporins (cefotaxime and cefmetazole). The susceptibility rates of IMP-6-positive K. pneumoniae for imipenem and meropenem were 100% and 75.0%, respectively.

**TABLE 2:** MIC range and MIC_50_ in carbapenemase-producing Klebsiella pneumoniae

Drug	MIC range (MIC_50_) (mg/liter)	
	IMP-1 producers	IMP-6 producers
Meropenem	0.25 to 64 (1)	0.25 to 16 (1)
Imipenem	0.125 to 64 (1)	≤0.06 to 0.5 (0.125)
Piperacillin	4 to 256 (128)	8 to >256 (>256)
Tazobactam/piperacillin	2 to 256 (64)	0.5 to 32 (8)
Cefotaxime	16 to 128 (64)	8 to >256 (64)
Ceftazidime	128 to >256 (>256)	4 to >256 (32)
Cefmetazole	256 to >256 (>256)	8 to >256 (64)
Aztreonam	≤0.06 to 1 (0.25)	≤0.06 to 128 (8)
Levofloxacin	≤0.06 to 32 (16)	≤0.06 to 128 (16)
Gentamicin	0.25 to 4 (1)	0.5 to 32 (2)
Amikacin	0.25 to 2 (1)	0.5 to 8 (2)

IMP-1 producers showed higher MICs toward ceftazidime and cefmetazole than IMP-6 producers, whereas the IMP-6 producers showed higher MICs toward aztreonam than the IMP-1 producers, which may be attributed to the coexpression of CTX-M enzymes.

### Transferability of resistance genes and plasmid incompatibilities.

Transconjugation experiments using E. coli J53 as the recipient identified transferable resistance genes in 85 isolates, with transfer frequencies ranging from 5.9 × 10^−8^ to 5.2 × 10^−3^ (Table 1); however, none of the isolates transferred virulence genes. Nine of the 21 (42.9%) IMP-1 producers transferred the resistance gene, whereas IMP-6 was transferred in 76 of the 83 (91.6%) IMP-6 producers. All 72 isolates coharboring IMP-6 and CTX-M-2 simultaneously transferred both genes, whereas the CTX-M-15 gene was not transferred in the IMP-6-, CTX-M-2-, and CTX-M-15-coharboring strain. Plasmid replicon typing revealed that 92 isolates (88.5%) harbored the IncN plasmid. IMP-1 producers showed various combinations of plasmid incompatibility, whereas most of the IMP-6 producers harbored only the IncN plasmid.

### Detection of carbapenem-resistant or hypervirulent STs among CPKp.

The detection of carbapenem-resistant or hypervirulent STs is shown in Table 3. Among the 104 CPKp isolates, five belonged to the CG258 non-*tonB79* cluster. However, no isolate belonging to the CG258-*tonB79* cluster was identified, including ST258.

**TABLE 3:** Sequence types, capsular types, and virulence genes in carbapenemase-producing Klebsiella pneumoniae

ST	No. of isolates	Capsular type		Virulence genes (no. of isolates)
		K1	K2	
CG258-*tonB79* cluster	0			
CG258 non-*tonB79* cluster	5	0	0	
ST23	4	4	0	*rmpA*, *rmpA2*, *iroN*, *iutA* (3)
ST65	12	0	12	*rmpA*, *rmpA2*, *iroN*, *iutA* (7); *rmpA*, *iutA* (1); *rmpA2*, *iutA* (2)
ST86	7	0	7	*rmpA*, *rmpA2*, *iroN*, *iutA* (1); *rmpA*, *rmpA2*, *iutA* (4); *rmpA2*, *iutA* (2)
Others	76	0	9	*rmpA*, *rmpA2*, *iroN*, *iutA* (8); *rmpA2*, *iroN*, *iutA* (1); *rmpA*, *rmpA2* (1); *rmpA*, *iroN* (2); *rmpA*, *iutA* (1); *rmpA2*, *iutA* (7); *iutA* (1)
Total	104	4	28	41

### Profiles of capsular types and virulence genes in CPKp.

All of the ST23 isolates had a K1 capsular serotype; however, this capsular type was not found in any other ST. The K2 capsular serotype was detected in 12 ST65 isolates, 7 ST86 isolates, and 9 other ST isolates (Table 3). Virulence genes were detected in 41 isolates (28 isolates with *rmpA*, 36 isolates with *rmpA2*, 22 isolates with *iroN*, and 38 isolates with *iutA*), and these genes were commonly found to be harbored in duplicates.

## DISCUSSION

In this study, we isolated 104 CPKp from clinical isolates throughout Japan. All isolates were found to produce IMP-type carbapenemase, and no isolate produced any other carbapenemases. Among these 104 IMP producers, 21 isolates were IMP-1 producers without a CTX-M gene, and 83 isolates were IMP-6 producers, the majority of which coharbored various CTX-M genes. In comparison with our previous report of carbapenemase-producing E. coli (5), there was a larger proportion of IMP-1 producers among CPKp, but only 9 of the 21 IMP-1 producers transferred the gene, suggesting that IMP-1 dissemination occurs in a clonal fashion or via a plasmid with a relatively narrow host range. In contrast, most of the IMP-6 producers coharbored CTX-M-2, and both the IMP-6 and CTX-M-2 genes were transferable with high frequency. Yamagishi et al. (15) reported IMP-6-carrying IncN plasmids isolated from 2013 to 2014 in Japan, including three strains of K. pneumoniae. In Japan, the IMP-6-carrying IncN plasmids may have been predominant since 2013. In this study, we found that 79 of the 83 IMP producers harbored the IncN plasmid. Among the 79 IMP-6-carrying IncN plasmids, 76 were transferable; IncN plasmid can spread to *Enterobacteriaceae*, especially E. coli and K. pneumoniae (16). These characteristics are similar to those of carbapenemase-producing E. coli, indicating that dissemination of the IMP-6 and CTX-M-2 genes mainly occurs via a plasmid with a broad host range such as the IncN plasmid (16, 17), highlighting the possibility of wide dissemination of these genes to other species.

Most metallo-β-lactamases, including IMP-6, have previously been detected in nonfermenting Gram-negative rods with low pathogenicity such as Pseudomonas aeruginosa and Acinetobacter spp. (18). It is noteworthy that the IMP-6-producing strains isolated in Japan have also been identified in relatively highly pathogenic bacteria such as E. coli (5). In this study, most of the IMP-6-producing K. pneumoniae isolates (83/104) simultaneously produced the CTX-M-type extended-spectrum β-lactamase. This means that monobactams (which are normally hard to degrade by metallo-β-lactamases) are susceptible to degradation by CTX-M-type enzymes, making β-lactams an unsuitable choice for the treatment of infections with IMP-6-producing strains.

In 2001, we reported the first isolation of an IMP-6-producing Serratia marcescens strain, which was detected in the urine sample of a Japanese patient with a urinary tract infection (18). Since then, the spread of this enzyme was not detected until Shigemoto et al. (17) reported the isolation of five IMP-6-producing strains of K. pneumoniae in Japan in 2012. In the same year, we detected 49 E. coli strains with IMP-6 and 5 strains with IMP-1 among sodium mercaptoacetic acid test (Eiken Chemical Co. Ltd., Tokyo, Japan)-positive strains collected throughout Japan (5). In this study, we confirmed a similarly high isolation rate of IMP-6 producers among K. pneumoniae as found for E. coli in Japan.

IMP-6 is a variant of IMP-1, in which adenine at nucleotide position 640 undergoes a mutation to guanine, resulting in substitution of serine by glycine at the 196th amino acid residue (18). This amino acid substitution causes a change in the substrate specificity for carbapenems. IMP-1 hydrolyzes imipenem more efficiently than meropenem, whereas IMP-6 hydrolyzes meropenem more effectively than imipenem. The current Clinical and Laboratory Standards Institute (CLSI) criteria for resistance of *Enterobacteriaceae* to carbapenems are as follows: MIC ≥ 4 μg/ml is resistant, 2 μg/ml is intermediate, and ≤1 μg/ml is sensitive (19). Because the MIC of imipenem for IMP-6-producing K. pneumoniae is only ≤0.06 to 0.5 μg/ml, these strains are assessed as sensitive according to the CLSI criteria, making it difficult for clinicians to identify them as metallo-β-lactamase-producing strains. In Japan, imipenem is often used as a representative carbapenem for susceptibility testing (5); therefore, IMP-6-producing isolates may be falsely categorized as susceptible if imipenem is employed in the panel of antimicrobial agents. In this study, the susceptibility rate of IMP-6-positive E. coli for meropenem was 75.0%, suggesting that several IMP-6-producers might have been miscategorized as susceptible to meropenem, like for imipenem. Therefore, establishing a laboratory screening method for these isolates using drugs other than imipenem and meropenem is essential.

Among the 104 CPKp isolates analyzed in this study, five isolates were found to belong to the CG258 non-*tonB79* cluster and did not harbor virulence genes. No isolates of the CG258-*tonB79* cluster or ST307 were detected. Isolates belonging to the CG258-*tonB79* cluster have been reported in the United States and Europe (20), ST307 has been identified in Europe and Africa (11, 12, 21), and the CG258 non-*tonB79* cluster was identified in East Asia, including China and Korea (20). Our data suggest that the CG258 non-*tonB79* cluster acquired *bla*_IMP_ and that the CG258 *tonB79* cluster and ST307 have not yet been imported to Japan. ST23, ST65, ST86, and ST375 were reported as hypervirulence-type K. pneumoniae (6, 8). In our study, 4 K1-ST23 isolates, 12 K2-ST65 isolates, and 7 K2-ST86 isolates were detected. Isolates showing that hypervirulence have previously been reported in Japan (9), and our data suggest the emergence of hypervirulence isolates among CPKp in Japan. In conjugation experiments with E. coli J53, none of these virulence genes was transferred. The nontransferability of virulence genes and transferability of resistance genes suggests that hypervirulence isolates have acquired resistance genes.

Carbapenemase-producing E. coli has previously been reported, while several studies had little or no carbapenemase-producing K. pneumoniae isolates in Japan. Therefore, the predominant carbapenemase and its molecular characteristics among K. pneumoniae isolates found in Japan have yet to be identified. We have collected over 100 isolates of carbapenemase-producing K. pneumoniae and demonstrated the molecular characteristics and epidemiology of IMP-6 producers coharboring various CTX-M genes in Japan. In conclusion, through analysis of the molecular characteristics of CPKp, the presence of isolates harboring both resistance and virulence genes was revealed. Moreover, our data suggest the acquisition of carbapenemase and CTX-M genes among high-virulence isolates, requiring further attention and countermeasures to control the spread of isolates with high virulence and antibiotic resistance.

## MATERIALS AND METHODS

### Bacterial strains.

From September 2014 to July 2015, 104 carbapenemase-producing K. pneumoniae isolates were randomly obtained from nonduplicate clinical isolates in 37 hospitals and outpatient clinics throughout Japan. The production of carbapenemase was confirmed by the carbapenem inactivation method (22). All strains were isolated from either infection or colonization/screening, and only one isolate per patient was included in this study. Among the 104 isolates, 42 were isolated from sputum, 40 from urine, 13 from feces, 2 from blood, and 7 from other sites.

### PCR detection and DNA sequencing of β-lactamase genes.

PCR was performed to detect carbapenemase genes, including *bla*_IMP_, *bla*_VIM_, *bla*_NDM_, *bla*_KPC_, and *bla*_OXA-48-like_ (23, 24), and CTX-M genes, including *bla*_CTX-M-1 group_, *bla*_CTX-M-2 group_, *bla*_CTX-M-9 group_, *bla*_CTX-M-8_, and *bla*_CTX-M-25_ (25). For positive isolates, PCR was performed for sequencing, and the products were sequenced on both strands with an ABI3730XL analyzer (Applied Biosystems, Foster City, CA, USA). BLAST version 2.2.24 (http://blast.ddbj.nig.ac.jp) was used to process the sequencing data and identify genes.

### Antimicrobial susceptibility testing.

The MICs of various antimicrobial agents were determined by the agar dilution method according to CLSI guidelines (19). Quality control for the MIC analysis was performed with E. coli ATCC 25922. Resistance to the following antibiotics was interpreted according to the breakpoints of CLSI guidelines (19); meropenem, ≥2 mg/liter; imipenem, ≥2 mg/liter; piperacillin, ≥32 mg/liter; tazobactam/piperacillin, ≥32 mg/liter; cefotaxime, ≥2 mg/liter; cefmetazole, ≥32 mg/liter; levofloxacin, ≥4 mg/liter; gentamicin, ≥8 mg/liter; amikacin, ≥32 mg/liter.

### Transferability of β-lactamase genes.

Transferability of β-lactamase genes was determined by conjugation experiments using the broth mating method as previously described (18). Exponential-phase lysogeny broth (LB) cultures of the donor strain and recipient strain E. coli J53 were mixed at a volume ratio of 1:1. This mating mixture was incubated overnight at 35°C. The transconjugants were selected on LB agar containing 100 mg/liter of sodium azide and 8 mg/liter of cefpodoxime.

### Plasmid incompatibility typing.

Plasmid incompatibility (Inc) groups were determined using the PCR replicon-typing scheme as previously described (26).

### Detection of STs associated with carbapenemase production and virulence.

For detection of carbapenem-resistant strains (CG258-*tonB79* cluster and non-*tonB79* cluster) and hypervirulent strains (ST23, ST65, ST86, and ST375), multiplex PCR was performed using specific primers as previously reported (8). For the detection of K. pneumoniae ST307, PCR was performed using the specific primer for ST307 (11).

### Detection of capsular genotype and virulence genes.

K1, K2, KL47, and KL64 isolates were identified with multiplex PCR as previously described (8). PCR was performed targeting the capsular polymerase genes *wzy*_K1_, *wzy*_K2_, *wzy*_KL47_, and *wzy*_KL64_ and the virulence genes *rmpA*, *rmpA2*, *iroN*, and *iutA* (8).
